# A new kind of beat

**DOI:** 10.7554/eLife.67701

**Published:** 2021-04-26

**Authors:** Kirsty Y Wan

**Affiliations:** Living Systems Institute, University of ExeterExeterUnited Kingdom

**Keywords:** flagellar beat, flagellar mechanics, extra-axonemal structures, euglena gracilis, flagella, Other

## Abstract

New mathematical model reveals how the flagella of some single-celled algae generate a lasso-like beat pattern that propels the cell through water.

**Related research article** Cicconofri G, Noselli G, DeSimone A. 2021. The biomechanical role of extra-axonemal structures in shaping the flagellar beat of Euglena gracilis. *eLife* **10**:e58610. doi: 10.7554/eLife.58610

Many organisms – ranging from single-cell protists to humans – rely on microscopic hair-like structures called cilia and flagella to perform a wide range of roles. In the human respiratory system, for example, large numbers of cilia work ceaselessly to remove dirt and pathogens from the lungs and airways (Sims et al., 2005): sometimes they move backwards and forwards in the plane, and sometimes they follow a more complicated trajectory (Figure 1A). Flagella are typically larger than cilia, and many single-cell organisms rely on the whip-like beating motion of flagella to propel them through the water in which they live.

**Figure 1.**
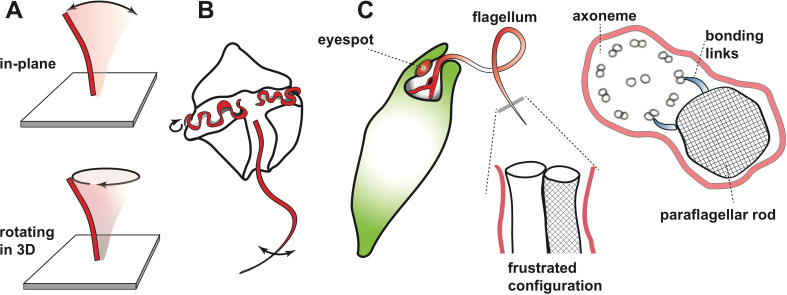
Beating motion of flagella and cilia. (**A**) Cilia and flagella stir fluid by moving back and forth in their own plane (top), or in a rotary fashion (bottom). (**B**) The flagella of single-celled organisms exhibit diverse beat patterns, sometimes on the same cell. For example, dinoflagellates have a transverse flagellum (red) which loops around the cell’s circumference and propagates helicoidal waves, and a longitudinal flagellum which moves back and forth behind the cell. (**C**) The alga *Euglena gracilis* has one short flagellum (inside the cell body), and one long flagellum, which contains a core axoneme (white) and a paraflagellar rod (lattice pattern). When the two incompatible structures ‘glue’ together via bonding links (right side image), this creates a mechanical frustration that forces both structures to bend together. As a result, the flagellum bends out of the plane and generates a lasso-like motion which pulls the alga along a helical path.

At the centre of most cilia and flagella is a structure called an axoneme, which consists of two central microtubules inside a circle formed of nine pairs of microtubules. Motor proteins, such as dynein, are able to bind to the microtubules and produce forces that cause the axoneme – and therefore the cilium or flagellum itself – to bend. A combination of mechanical feedback and active signalling coordinates the actions of the motor proteins, and through them, the overal pattern of the beating motion. Different organisms rely on different variations of this basic mechanism, with the greatest variety being found in protists (presumably because one billion years of evolution has provided them with ample opportunity to evolve highly-specialised adaptations to many environmental niches).

Single-celled organisms can have multiple flagella that can differ (sometimes dramatically) in length and/or shape, and even beat at different frequencies (Figure 1B). For example, an alga called *Euglena gracilis* has two flagella, including one that whirls around like a propeller or lasso in front of the cell and pulls it through the water along a helical path (Leedale et al., 1965). Many protists use this universal strategy to scan their environment (Cortese and Wan, 2021), but the mechanism underlying the lasso-like motion of flagella in *Euglena* has remained a mystery. Now, in eLife, Giancarlo Cicconofri, Giovanni Noselli and Antonio DeSimone – who are based at the Centre Internacional de Mètodes Numèrics a l’Enginyeria, International School for Advanced Studies (SISSA) and Scuola Superiore Sant’Anna – report how interactions between the axoneme and an unusual structure called the paraflagellar rod may be responsible (Cicconofri et al., 2021). The paraflagellar rod is only found in certain protist groups, where it runs parallel to the axoneme and has a similar diameter (Figure 1C). Moreover, it attaches to specific locations on the axoneme via elastic bonding links, which suggests that it has a role in motility (Melkonian et al., 1982).

Cicconofri et al. developed a detailed mathematical model of the flagellum that replicated the contrasting physical properties of the paraflagellar rod and axoneme. The model showed that when the paraflagellar rod and axoneme ‘glue’ together to form a single structure, the resulting mechanical frustration can cause the axoneme to bend out of plane (Figure 1C). The researchers then go on to show that when dynein motor proteins move in a way that causes a simple planar motion in the absence of the paraflagellar rod, this instead generates a three-dimensional lasso-like motion and helical swimming when the axoneme and paraflagellar rod are glued together. By uncovering a connection between the paraflagellar rod and flagellar motility, Cicconofri et al. may have arrived at a general principle for the generation of complex spatiotemporal dynamics: keeping the underlying control principles simple and harnessing mismatched mechanical properties.

Plants also exploit this approach to achieve surprising levels of speed and agility. For instance, the hair-like awn of *Erodium* plant seeds are wrapped in stiff cellulose fibres arranged in a tilted helical pattern, which help disperse the seed by uncoiling and coiling the awn in response to humidity (Geer et al., 2020). A similar intimate coupling exists between the internal flagellum and cell body of some spirochaete bacteria, which gives the bacteria their helical shape and determines their movement and virulence (Dombrowski et al., 2009).

Like many other single-celled organisms, *Euglena* swim along helical paths to scan their local environment (Rossi et al., 2017). In the case of *Euglena* this is augmented with photoreception, so that the alga can navigate efficiently towards or away from light for photosynthesis. It is possible that the paraflagellar rod can actively deform and alter the beat pattern of the flagellum based on light intensity (Piccinni, 1975), perhaps in combination with a photoreceptor located at its base (Rosati et al., 1991). The mathematical model developed by Cicconofri et al. could be used to test this hypothesis.

Major open questions still remain, such as how and when the paraflagellar rod evolved, and whether it arose in a similar way in different species (Lukes et al., 2009)? For example, the paraflagellar rod is also found in closely-related organisms called kinetoplastids, which includes several parasitic flagellates that cause infectious diseases in humans. *Euglena* is a useful and versatile model system for answering these questions. The curious features that *Euglena* and other single-celled organisms evolved over billions of years could be applied to the generation of intelligent materials in soft robotics. This goes to show that no matter whether you are a mathematician or a biologist, nature continues to inspire.
